# Ongoing Slow Fluctuations in V1 Impact on Visual Perception

**DOI:** 10.3389/fnhum.2016.00411

**Published:** 2016-08-23

**Authors:** Afra M. Wohlschläger, Sarah Glim, Junming Shao, Johanna Draheim, Lina Köhler, Susana Lourenço, Valentin Riedl, Christian Sorg

**Affiliations:** ^1^Department of Neuroradiology, Technische Universität MünchenMunich, Germany; ^2^TUM-Neuroimaging Center, Technische Universität MünchenMunich, Germany; ^3^Graduate School of Systemic Neurosciences, Ludwig-Maximilians-Universität MünchenMartinsried, Germany; ^4^Department of Computer Science and Technology, University of Electronic Science and Technology of ChinaChengdu, China; ^5^Department of Nuclear Medicine, Technische Universität MünchenMunich, Germany; ^6^Department of Psychiatry, Technische Universität MünchenMunich, Germany

**Keywords:** slow fluctuations, ongoing BOLD signal, pre-stimulus activity, visual consciousness, backward masking

## Abstract

The human brain’s ongoing activity is characterized by intrinsic networks of coherent fluctuations, measured for example with correlated functional magnetic resonance imaging signals. So far, however, the brain processes underlying this ongoing blood oxygenation level dependent (BOLD) signal orchestration and their direct relevance for human behavior are not sufficiently understood. In this study, we address the question of whether and how ongoing BOLD activity within intrinsic occipital networks impacts on conscious visual perception. To this end, backwardly masked targets were presented in participants’ left visual field only, leaving the ipsi-lateral occipital areas entirely free from direct effects of task throughout the experiment. Signal time courses of ipsi-lateral BOLD fluctuations in visual areas V1 and V2 were then used as proxies for the ongoing contra-lateral BOLD activity within the bilateral networks. Magnitude and phase of these fluctuations were compared in trials with and without conscious visual perception, operationalized by means of subjective confidence ratings. Our results show that ipsi-lateral BOLD magnitudes in V1 were significantly higher at times of peak response when the target was perceived consciously. A significant difference between conscious and non-conscious perception with regard to the pre-target phase of an intrinsic-frequency regime suggests that ongoing V1 fluctuations exert a decisive impact on the access to consciousness already before stimulation. Both effects were absent in V2. These results thus support the notion that ongoing slow BOLD activity within intrinsic networks covering V1 represents localized processes that modulate the degree of readiness for the emergence of visual consciousness.

## Introduction

Intrinsic brain networks, characterized by coherent patterns of slowly fluctuating ongoing activity, constitute a fundamental organization principle of the human brain (Damoiseaux et al., 2006; Raichle, 2010). In the last decade, a wealth of studies has shown an association between such patterns of ongoing brain activity and the brain’s normal and, in case of pathology, abnormal functions (Greicius et al., 2004; Biswal et al., 2010; Smith et al., 2012; Fox et al., 2014). As such networks have traditionally been investigated in the resting state, however, there is still a lack of understanding regarding the underlying processes and their relevance for human cognition, behavior, and perception (see Keilholz, 2014, for a review). To address this point, the present study focuses on ongoing blood oxygen level dependent (BOLD) activity in occipital areas V1 and V2 and its relevance for emerging conscious visual perception.

Evidence for the behavioral relevance of ongoing BOLD activity comes from studies of behavioral variability in the face of invariant task requirements. The contribution of ongoing activity at about 0.1 Hz to trial-to-trial variability in motor responses, for example, has been successfully demonstrated by Fox et al. (2007). These authors showed that fluctuations of activity in the ipsi-lateral somatomotor cortex during button presses account for a significant fraction of trial-to-trial variability in evoked contra-lateral BOLD responses. Importantly, those fluctuations were also found to predict fluctuations in right-hand button press strength. Several studies provide similar results from the analysis of trial-to-trial variability in BOLD activity by showing direct relations to variability in cognition, behavior, or perception, regarding for example ratings of pain intensity or performance in a working memory task (Pessoa et al., 2002; Boly et al., 2007; Fox et al., 2007; Eichele et al., 2008).

Such a relevance of ongoing BOLD activity has also been shown with regard to variability in visual processing. The correct as compared to the incorrect perception of visual gratings, for example, has been associated with higher ongoing BOLD activity at the peak time of the BOLD response in the stimulated V1 (Schölvinck et al., 2012). Interestingly, studies of higher visual areas have shown that the access to visual consciousness can be predicted already before stimulation. Pre-stimulus activity in the fusiform face area, for example, was found to predict face perception for Rubin’s ambiguous face-vase pictures (Hesselmann et al., 2008a). Likewise, pre-stimulus activity in the right occipito-temporal cortex was found to be higher when coherent visual motion in a dynamic random dot display was detected as compared to missed (Hesselmann et al., 2008b).

More hints at the impact of pre-stimulus brain states on visual perception have been provided by electroencephalographic (EEG) studies. Busch et al. (2009), for example, showed that the detection probability of at-threshold stimuli is dependent on the pre-stimulus phase in the theta and alpha bands. Furthermore, pre-stimulus slow cortical potentials (SCPs), reflecting frequencies below 0.1 Hz, were found to be more negative in trials of perceived stimuli as opposed to trials of non-perceived stimuli (Devrim et al., 1999). Interestingly, such slow fluctuations in electrophysiological activity seem to be systematically related to ongoing BOLD activity, particularly within intrinsic brain networks (He and Raichle, 2009; Schölvinck et al., 2013; Hiltunen et al., 2014).

To this end, we propose that ongoing BOLD activity in early visual areas immediately before stimulation as well as at the peak time of stimulus-evoked activity impacts on the emergence of conscious visual perception. In our study, perception was assessed on a trial-to-trial basis in a backward masking paradigm, using subjective ratings of confidence as a proxy for consciousness. This operationalization followed previous recommendations (Dehaene and Changeux, 2011) and has been successfully employed in imaging studies before (Lau and Passingham, 2006; Britz et al., 2014). By stimulating exclusively one visual hemifield, only one brain hemisphere experienced direct input from the visual task paradigm. Ongoing BOLD activity in brain regions of active visual processing (i.e., contra-lateral) could therefore be approximated by activity in functionally connected voxels of the non-stimulated (i.e., ipsi-lateral) hemisphere (Fox et al., 2007; Schölvinck et al., 2012). Selected voxels were taken from two bilateral intrinsic connectivity networks (ICNs) in the early visual cortex, which were derived from spatial high-resolution resting-state functional magnetic resonance imaging (fMRI) and could be associated with visual areas V1 and V2 respectively. In a first analysis, we assessed whether conscious visual perception is associated with the ipsi-lateral BOLD signal magnitude after (see Schölvinck et al., 2012) as well as before target presentation. As visual perception has been proposed to operate in periodic cycles of alternating cortical excitability and cortical inhibition (Busch et al., 2009), we subsequently band-pass filtered the ipsi-lateral BOLD activity to the peak frequency of an additional resting-state fMRI run, thereby getting rid of any remaining task effects in ipsi-lateral voxels, and assessed whether this signal’s phase affects an imminent target’s access to consciousness.

## Materials and Methods

### Ethical Approval

All procedures performed in this study were in accordance with the ethical standards of the TUM School of Medicine of the Technical University of Munich and with the 1964 Helsinki declaration and its later amendments or comparable ethical standards.

### Participants and Task

Sixteen healthy participants (21–28 years old, 11 females) took part in this study. Informed consent was obtained from all participants. All participants had normal or corrected-to-normal vision.

**FIGURE 1:**
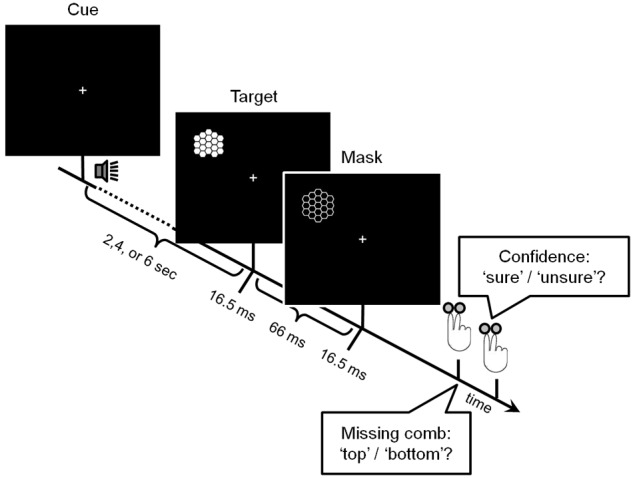
**Experimental paradigm.** Each trial started with an auditory cue, which carried information about the target’s location in half of the runs. After a variable interval of 2, 4, or 6 s, the target -a honeycomb pattern with one missing comb either at the top or at the bottom- appeared for 16.5 ms. After another 66 ms, a negative image of the complete pattern was presented for 16.5 ms, serving as the mask. Both target and mask appeared exclusively in the left visual field. Participants had to report in two binary-choice questions, first, which comb was missing and, second, whether they were sure or had guessed the answer.

The backward masking paradigm is summarized in **Figure 1**. Stimuli for target and mask were comparable to those used by Haynes et al. (2005). Targets consisted of a honeycomb pattern in which either the top or the bottom comb was missing. A subsequently presented negative image of the complete honeycomb pattern served as the mask. Participants had to report in two binary-choice questions, first, the location of the missing comb and, second, whether they were sure or guessed the answer. Confidence therefore was used as a proxy for the access to consciousness on a trial-to-trial basis (Lau and Passingham, 2006; Dehaene and Changeux, 2011; Britz et al., 2014). Timing parameters were chosen such that masking produced error rates of above 30% on average, which was extensively tested in behavioral experiments previous to the study. The center of the screen was marked by a fixation cross. Participants were asked to maintain fixation throughout the task, which was monitored by an eye-tracker. Importantly, to enable the measurement of a proxy of early visual cortex ongoing activity during visual stimulation, targets were shown exclusively in the left visual field. Stimulation pseudo-randomly occurred at two positions either in the upper or in the lower left quadrant, covering visual angles between 7° and 10° outside foveal vision. To ensure that stimulation of the occipital system was not affected by preceding trials, the inter-trial interval (ITI) between two targets was 28 ± 6 s (mean ± standard deviation, median: 28 s, minimum: 14 s, maximum: 38 s). A sound cue (monotone, 25 ms) was presented before the target to ensure that participants fixated the central cross after these relatively long ITIs. The cue occurred at three different cue-target stimuli onset asynchronies (SOAs) of 2, 4, and 6 s to modulate temporal attention. Spatial attention was modulated by either indicating the stimulation quadrant via the cue tone’s pitch in half of the runs or by providing no information via a neutral, intermediate pitch in the other half. Attentional effects are not evaluated here but were controlled for in the analyses.

Stimuli were presented using the Presentation Software (NeuroBehavioral Systems^1^) and the VisuaStim Digital goggle system (Resonance Technology, Inc.^2^) with an integrated ViewPoint EyeTracker system (Arrington Research, Inc.^3^).

In total, the visual perception task consisted of four runs. Each run had a duration of 15.5 min and contained 20 targets, resulting in 13.3 events per attention condition and participant before the exclusion of events due to ratings from the eye-tracker data evaluation. Participants underwent one training session of the paradigm in the week before scanning and were familiar with the task and the cue tones.

### Behavioral Data Analysis

#### Eye-Tracker Data

A box with a diagonal of 7° was created around the center of the screen. Signal time courses of trials were only included in the analyses if the gaze position was within this box at the time point of target presentation. The median of the number of trials removed per participant was 1 (minimum: 0, maximum: 25).

#### Button Presses

The effect of backward masking was validated by testing (i) if the percentage of the response ‘sure’ was substantially lower than 100% and (ii) if the error rate was increased for events labeled ‘unsure’ as compared to events labeled ‘sure.’

In order to verify that the variable ITI, necessary for the analysis of BOLD effects, did not introduce systematic behavioral effects, we analyzed the dependence of both response classes (‘correct’/‘incorrect’ and ‘sure’/‘unsure’) on the ITI with two different procedures: For each class, we first compared the median ITI per participant between the two response options with a Wilcoxon signed rank test. Second, we calculated regressions of the overall fraction of ‘correct’ responses and ‘sure’ responses on the 11 ITI values realized by the design.

### Imaging Data Acquisition

Imaging data were acquired on a Philips 3T Achieva Quasar Dual with an 8-channel phased-array head coil. Data consisted of the following sequences: (i) To define visual ICNs, 6 min of spatial high-resolution resting-state fMRI with 240 echo planar imaging (EPI) scans and a limited field of view focusing on the occipital cortex was conducted (TE = 35 ms, TR = 1500 ms, flip angle = 82°, FoV = 196 mm × 48 mm × 220 mm, matrix = 112 × 108, 22 slices, slice thickness = 2 mm, and 0.2 mm interslice gap). (ii) To realize the backward masking task, we performed four runs of 460 whole-brain gradient EPI scans of 15.5 min each (TE = 35 ms, TR = 2000 ms, flip angle = 82°, FoV = 220 mm × 128 mm × 220 mm, matrix = 80 × 80, 32 slices, slice thickness = 4 mm, and no interslice gap). (iii) To control for potential task design influences on ongoing BOLD activity (see Section “Frequency-Selective Analysis of the BOLD Phase”), additional 6 min of whole-brain resting-state fMRI were collected [for parameters, see (ii)]. (iv) To co-register the fMRI data, a high-resolution T1-weighted anatomical scan was recorded for each participant (TE = 4 ms, TR = 9 ms, TI = 100 ms, flip angle = 5°, FoV = 240 mm × 240 mm, matrix = 240 × 240, 170 slices, voxel size = 1 mm × 1 mm × 1 mm).

### Definition of ROIs in the Occipital Cortex

#### Definition of icnV1 and icnV2 from High-Resolution Resting-State fMRI

**FIGURE 2:**
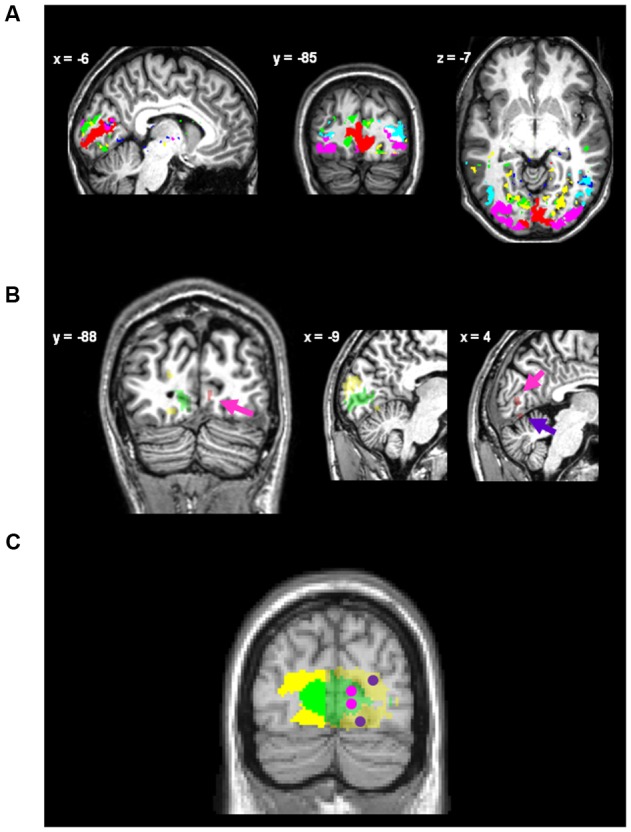
**Regions of interest.** **(A)** The occipital intrinsic networks defined via back-reconstruction after ICA on spatial high-resolution resting-state fMRI data for a randomly selected single participant, superimposed onto this participant’s normalized anatomical image. Networks were identified as posterior icnV1 (red), anterior icnV1 (blue), icnV2 (green), icnV3 (magenta), icnV4 (yellow), and icnV5 (turquoise). **(B)** Regions of interest (ROIs) for data extraction of one single participant. Coordinates are given in MNI space (for visual inspection only as non-normalized data is depicted). The ipsi-lateral ROIs for icnV1 (green) and icnV2 (yellow) are depicted along with the activation peaks within the contra-lateral hemisphere (red) for upper left visual quadrant stimulation. Magenta and purple arrows indicate the attribution of these peaks to icnV1 and icnV2, respectively. An overlay of the contra-lateral ROIs for stimulation of the upper and the lower visual quadrant within V1 (magenta) and V2 (purple) with networks icnV1 (green) and icnV2 (yellow) is shown in **(C)**, constructed from the average of the single-subject ROIs (normalized to MNI standard space for display purposes only). Voxels within 6 mm of the interhemispheric gap were not included in the ROIs to avoid carry-over effects caused by spatial smoothing of the data. The parts of the networks included as ipsi-lateral ROIs are opaque.

Two bilateral functional connectivity maps were characterized and associated with V1 and V2 via regional overlap with cytoarchitectonical templates. The exact procedure was as follows:

High-resolution resting-state fMRI data were preprocessed using statistical parametric mapping (SPM5; Wellcome Trust Centre for Neuroimaging^4^). Preprocessing included slice-timing and motion correction, co-registration to the whole-brain EPI series, spatial normalization, and spatial smoothing with a 2 mm × 2 mm × 2 mm Gaussian kernel (for more details concerning high-resolution resting-state fMRI data analysis, see Pasquini et al., 2014). A voxel-wise Z-transformation was applied to the time course data to render sensitivity for variance correlation independent of variance magnitude. Subsequently, data were entered into a group-level independent component analysis (ICA) in GIFT (Medical Image Analysis Lab, The MIND Research Network^5^). Out of 35 spatially independent components (IC), six ICs had an activity pattern centered in the occipital cortex. **Figure 2A** shows the spatial distribution of back-reconstructed individual occipital ICs for a randomly selected single participant.

Using the Anatomy Toolbox for SPM (Eickhoff et al., 2005), we assessed the spatial overlap of these ICs with cytoarchitectonically defined occipital areas hOC1, hOC2, hOC3v, hOC4, and hOC5, which correspond well with the functionally defined retinotopic areas V1, V2, V3v, V4, and V5 (Wohlschläger et al., 2005; Wilms et al., 2010). With respect to regions of interest, two ICs covered hOC1/V1 (amount of IC1 covering the area: 78%; IC2: 52%), while one IC overlapped substantially with hOC2/V2 (IC3: 49%). IC1 overlapped with posterior parts and IC2 with anterior parts of hOC1/V1. Such a distinction of anterior and posterior parts of early visual areas by spatial ICA on fMRI data has been described previously (Smith et al., 2012). Since we did not find significant task-associated activity in the contra-lateral IC2, we focused on IC1 and IC3 as surrogates for icnV1 and icnV2.

#### Generation of Individual icnV1 and icnV2 Maps

By re-normalization and thresholding of the ICA-derived maps, binary bilateral maps of icnV1 and icnV2 were constructed for each participant:

Individual participant images were back-reconstructed, masked (Tzourio-Mazoyer et al., 2002) and morphed into single-subject space using the inverse of the original normalization transformation. For each participant, maps of icnV1 and icnV2 were constructed by only including voxels that deviated positively from the image mean by more than 1.5 standard deviations. Image erosion and size thresholding steps created largely contiguous masks with minimal overlap. Remaining overlapping regions between masks were attributed to the mask with the highest *z*-score before binarization. These maps served as a common basis for the construction of regions of interest (ROIs) in the ipsi-lateral (non-stimulated) as well as in the contra-lateral (stimulated) hemisphere.

#### Localization of Ipsi-Lateral Occipital Sites within icnV1 and icnV2

Only the left hemispheric parts of icnV1 and icnV2 were used as ROIs for the extraction of the ipsi-lateral signal. Approximate size and position of the resulting ROIs is indicated by the opaque parts in **Figure 2C**. The exact procedure was as follows:

IcnV1 and icnV2 maps were masked by a left hemisphere mask including only areas more than 6 mm to the left of the inter-hemispheric gap to rule out carryover effects of signal smeared out from the contra-lateral hemisphere. The resel-size estimate of the general linear model (GLM) analysis with SPM on the smoothed images was 6 mm × 6 mm × 6 mm. The size of the ROIs in units of voxels (number of signal time courses, voxel size = 2.3 mm × 2.3 mm × 4 mm) was: icnV1: 113 ± 52 (mean ± standard deviation)/111 (34-205) [median (minimum–maximum)], icnV2: 143 ± 46/143 (61–219).

#### Localization of Contra-Lateral Occipital Sites within icnV1 and icnV2

Sites of significant activation by the target within icnV1 and icnV2 were detected in each participant and small spherical ROIs were created. Approximate size and position of the resulting ROIs is also indicated in **Figure 2C**.

First, MNI-coordinates of significant activation by the target covering all retinotopic areas were defined in each participant. To do this, task-fMRI data were preprocessed using SPM including motion correction, co-registration of the anatomical image, and spatial smoothing with a 4 mm × 4 mm × 4 mm Gaussian kernel. A GLM analysis in single-subject space was performed including regressors coding for stimulation in the lower and upper visual quadrant as well as their temporal derivatives to allow for shifts in the timing of the BOLD response peak. T-maps of the contrasts between the stimulation quadrants were combined with those of the contrasts of the respective derivatives by a maximum operation.

Second, peaks within icnV1 and icnV2 were selected. For this, four peak coordinates of stimulus-related activity were determined, within the two areas icnV1 and icnV2 and at the two sites for upper and lower visual field stimulation. To do this, the combined *t*-value maps of the stimulation quadrant contrasts were masked by the binary maps of icnV1 and icnV2 and a right hemisphere mask for each participant. After automatically finding the coordinates of highest *t*-values, the positions super-imposed onto the anatomical images of the participants were checked by three raters with the following criteria: (i) Activation for upper visual field stimulation should be located in the lower occipital cortex and vice versa. (ii) Activation attributed to V1 should be located in the grey matter of the calcarine sulcus. Overall, 23 out of 128 peak coordinates were replaced by the coordinates of the second highest peaks. To further assure that activation was actually related to the stimulation, only those peaks were included into further analyses which had a *t*-value > 3.73 (p_u_ < 0.0001). This threshold was more liberal than p_SVC_ < 0.05, small volume corrected for right icnV1 or icnV2, respectively. If the *t*-value was lower, the location was not accepted as a stimulation position for the respective retinotopic area and participant and was excluded from task-ROI-analyses. ROIs of 4 mm radius were created. It has to be noted that contra-lateral ROIs were only about 1/10th of the size of ipsi-lateral ROIs.

### ROI-Based fMRI Data Analysis: Processing of Time Courses

BOLD signal time courses were extracted from all ROIs, filtered, and converted to percent signal change:

Signal time courses of preprocessed but non-smoothed task-fMRI data were extracted from the two ROIs of the ipsi-lateral hemisphere, the four ROIs of the contra-lateral hemisphere, and from individual white matter and cerebrospinal fluid (CSF) masks for the construction of confound regressors. For each time course, the mean signal *y* across the ROI was computed by averaging all values within the ROI at a given time point. *y* was translated into percent signal change *y*_PSC_, with *y*_PSC_ = (*y* – *b*)/*b*^∗^100. *b* was calculated as a run average over the last several time points starting 14 s after target presentation until the next target presentation. Equally transformed white matter and CSF signals were regressed out and the signal was high-pass filtered to frequencies above 128 s wave length.

### Event-Related Analysis of the BOLD Magnitude

#### Ipsi-Lateral Activity and the Access to Consciousness

Event-related time courses were constructed for each target onset by taking into account 2 TRs (4 s) before and 7 TRs (14 s) after target presentation. The relationship between the BOLD signal in the ipsi-lateral ROIs and conscious visual perception of the target was assessed pre- and post-target presentation. For this, data of 2 and 4 s before (PRE-stimulus) or after (PEAK-response) target presentation were averaged and used as dependent variables in the statistical models. Concerning the PEAK-response definition, one should note that a period of about 4 s after target presentation coincides with the peak of the BOLD response found in this study in the contra-lateral hemisphere and corresponds to the typical temporal delay of BOLD responses to stimulation (Bandettini et al., 1992; Logothetis, 2002; Buxton et al., 2004). The factor of interest, ‘conscious visual perception,’ was determined directly from participants’ second button press (‘sure’/‘unsure’), but trials which were rated as ‘sure’ with nevertheless wrongly detected targets were excluded from the analysis. Also excluded were those trials with negative eye-tracker ratings.

As changes of BOLD percent signal change in the occipital cortex are assumed to be comparable across participants, we chose a fixed-effects approach. Overall, this resulted in four analysis of variance (ANOVA) models for the ipsi-lateral ROIs: ‘icnV1 PRE-stimulus’ [degrees of freedom (df) = 905], ‘icnV1 PEAK-response’ (*Id.*), ‘icnV2 PRE-stimulus’ (*Id.*), and ‘icnV2 PEAK-response’ (*Id.*). *P*-values reported are Bonferroni-corrected by the number of ANOVAs, i.e., by a factor of 4. As a control analysis for unexpectedly high impact of between-subject variability, an additional analogous mixed-effects analysis was performed on the data, employing the same dependent variables, the same factors of interest, and the same factors of no-interest, which are described in the following paragraph.

In order to account for effects of different kinds of cueing, attentional conditions were modeled as independent variables along with the factor of interest. The model consisted of factors (i) ‘conscious visual perception’, (ii) temporal attention (at three levels with cue 2, 4, or 6 s before target), and (iii) spatial attention (spatial information in the cue present or absent). Pair-wise interactions of the factors were calculated. There were on average 76.3 trials per condition (median: 79.5, minimum: 50, maximum: 116).

#### Contra-Lateral Activity and the Access to Consciousness

For an analysis of the relationship of task-related BOLD signals and conscious visual perception, an entirely analogous procedure with ROIs in the contra-lateral hemisphere was performed. Here, an additional regressor was included in the models, coding the visual quadrant of stimulation (‘upper left’ or ‘lower left’). Again, we created four ANOVA models: ‘icnV1 PRE-stimulus task’ (df = 390), ‘icnV1 PEAK-response task’ (*Id.*), ‘icnV2 PRE-stimulus task’ (df = 724), and ‘icnV2 PEAK-response task’ (*Id.*). *P*-values reported are Bonferroni-corrected by a factor of 4. Df were smaller than in the analysis of the ipsi-lateral signal because the additional regressor coding for position of stimulation was used and because the task activation peak could not be detected reliably in some of the participants, resulting in smaller trial numbers. Average trial numbers per condition were for icnV1: 16.7 (median: 14.5, minimum: 8, maximum: 29) and for icnV2: 30.6 (median: 32, minimum: 14, maximum: 63).

Finally, a correlation of PRE-stimulus ipsi-lateral signal and PEAK-response contra-lateral signal was analyzed using Pearson’s correlation analysis. The correlation was calculated within each participant for whom a significant task peak had been detected. Significance was calculated across the group via *t*-tests on the Fisher-Z-corrected correlation coefficients.

### Frequency-Selective Analysis of the BOLD Phase

To assess the signal phase in a frequency-selective way, we analyzed the frequency spectrum of the BOLD signal within ipsi-lateral icnV1 and icnV2 from the whole-brain resting-state as well as the task-fMRI runs. From the resting-state spectrum, we derived the frequency of peak power. Using the task data, we then calculated the signal phase immediately before target presentation selectively at the peak frequency of the resting-state spectrum.

More specifically, time courses of the whole-brain rest-fMRI run were created in an entirely analogous procedure from the same ipsi-lateral ROIs as used in the task-fMRI runs. Time courses of the four task runs and the rest run were then entered as a whole-per-session into a Fourier analysis (MATLAB^6^). For both the task runs and the rest run, frequency spectra were created separately and averaged across participants (and runs, in case of the task-fMRI). For the rest-fMRI spectra of icnV1 and icnV2, the position of the absolute amplitude peak was then determined. A tight frequency band of 5^∗^10^-3^ Hz width around this peak (‘intrinsic-frequency regime’) was constructed and signal time courses of all task runs were band-pass filtered to this regime by a fifth-order Butterworth filter. The derived time courses were subjected to a Hilbert transform to construct time courses of the signal phase. The phase one TR before target presentation within this ‘intrinsic-frequency regime’ was averaged per participant, ROI, and consciousness category. For ipsi-lateral icnV1 and icnV2 separately, it was finally tested with a Wilcoxon signed rank test whether there were significant phase differences between consciousness categories.

The task frequency peak was known to be at 0.0334 Hz and turned out to be associated with the highest amplitude in the task-fMRI spectra of ipsi-lateral icnV1 and icnV2. The occurrence of qualitatively similar effects in this ‘task-frequency regime’ and the ‘intrinsic-frequency regime’ would indicate that bands might not have been sufficiently separated. In that case, possible effects related to signal phase might have been caused by the paradigm and related external attentional effects. To control for this, an entirely identical analysis as described above was performed for signals which were analogously band-pass filtered to the ‘task-frequency regime.’

Additionally, it was tested whether the phase was dependent on any of the attentional cue types by mutual Wilcoxon signed rank tests.

## Results

### Intrinsic Connectivity Networks in the Occipital Cortex

Spatial high-resolution resting-state fMRI and data-driven ICA revealed ICNs in the occipital cortex which largely overlapped with anatomical maps of retinotopic areas V1–V5 (**Figure 2A**). This result is in line with previous findings about the intrinsic organization of the occipital cortex (Cohen et al., 2008; Kiviniemi et al., 2009; Allen et al., 2011; Craddock et al., 2012; Dawson et al., 2013).

### Behavioral Results

**FIGURE 3:**
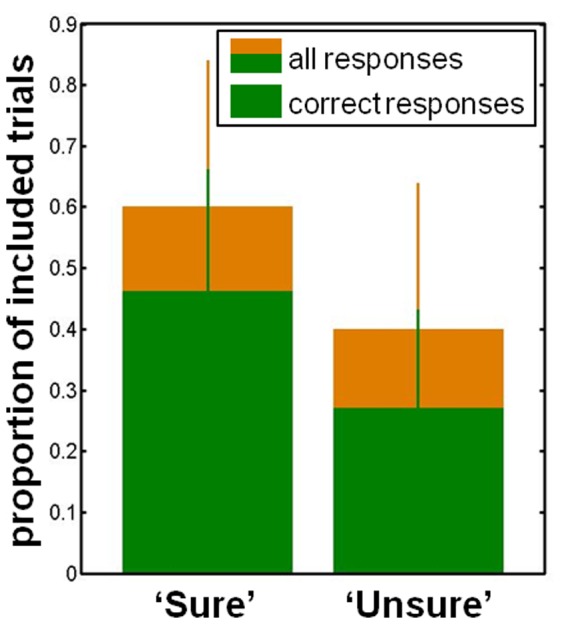
**Behavioral data.** The proportion of trials in which participants were ‘sure’ as well as those in which they were ‘unsure.’ The proportion of correct responses is shown in green for each consciousness category. Median and standard deviation are displayed.

We analyzed behavioral responses in order to assure that our realization of backward masking was effective. As can be seen in **Figure 3**, participants gave correct responses on the relevant target property in 76 ± 15% (median ± standard deviation) of trials and reported to be sure in 60 ± 24% (median ± standard deviation) of trials. There were significantly more correct responses when participants reported being sure than when they were uncertain (‘sure’ 84% (median), ‘unsure’ 65% (median), *p* < 0.001, *N* = 16, Wilcoxon signed rank test). The backward masking task thus efficiently enabled the desired manipulation of visual consciousness.

In order to rule out that the variation of ITIs introduced systematic behavioral effects, we next analyzed the dependence of responses on ITI length. With regard to task performance (‘correct’/‘incorrect’), we found no significant difference of the median ITI per participant between responses (*p* = 0.162, *N* = 16, Wilcoxon signed rank test). A regression of the fraction of correct responses against ITI yielded no significant correlation (*R* = 0.22, *F* = 0.45, *p* = 0.521, *N* = 11). Similarly, there was no significant difference (*p* = 0.609, *N* = 16, Wilcoxon signed rank test) or dependence (*R* = 0.24, *F* = 0.53, *p* = 0.484, *N* = 11) for responses ‘sure’/‘unsure.’ The variability of trial spacing in time did therefore not impact significantly on behavioral responses.

There was a significant effect of the attention conditions, *p* < 0.001 for spatial attention, and *p* = 0.026 for temporal attention, with no significant interaction (*p* = 0.120) on the rating ‘sure’ vs ‘unsure’.

### Ipsi-Lateral BOLD Magnitude in icnV1 is Associated with Access to Visual Consciousness

**FIGURE 4:**
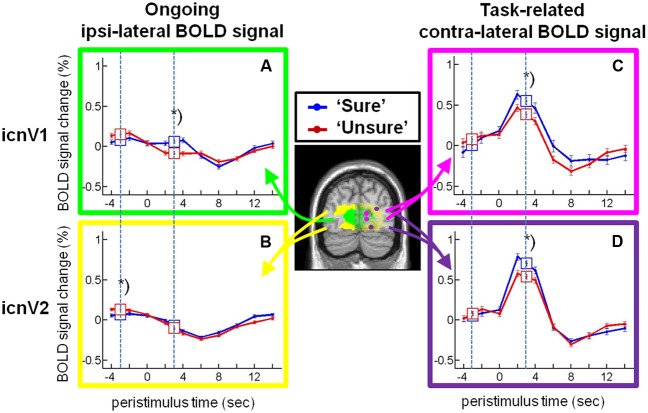
**Stimulus-locked activity in the intrinsic connectivity networks (ICNs) covering V1 and V2.** Time courses (mean and standard error of the mean) of the blood oxygenation level dependent (BOLD) percent signal change for categories ‘sure’ (blue) and ‘unsure’ (red) from **(A)** the ipsi-lateral icnV1, **(B)** the ipsi-lateral icnV2, **(C)** the contra-lateral icnV1, and **(D)** the contra-lateral icnV2 are plotted. Data were corrected for the effects of temporal attention, spatial attention, and stimulated visual quadrant. The time windows for which statistical tests were performed are indicated by squares containing the respective means and standard errors of the mean and by dashed lines. The asterisks label significant differences at *p*_c_ < 0.05 for ANOVA.

Next, we examined the relationship between the BOLD magnitude in the ipsi-lateral ROIs and conscious visual perception on a trial-to-trial basis (all *F*- and *p*-values assembled in **Table 1**). As can be seen in **Figures 4A,B**, the BOLD magnitude in icnV1 as well as in icnV2 prior to stimulation was lower when participants consciously perceived the target, reaching significance only in icnV2 (*p* = 0.048, Bonferroni-corrected). At the time of PEAK-response, the BOLD magnitude was significantly higher for consciously perceived targets in icnV1 (*p* < 0.001, Bonferroni-corrected).

**Table 1:** Ipsi-lateral BOLD activity in V1 and V2.

	Ipsi-V1 (df = 905)				Ipsi-V2 (df = 905)			
		
	PRE		PEAK		PRE		PEAK	
				
	*F*	*p*	*F*	*p*	*F*	*p*	*F*	*p*
Access to consciousness (AC)	3.44	0.064	**16.74**	**<0.001**	**6.33**	**0.012**	1.61	0.206
Temporal attention (TA)	2.58	0.077	**40.37**	** <0.001**	0.15	0.861	**12.75**	** <0.001**
Spatial attention (SA)	2.28	0.132	0.79	0.373	0.53	0.465	1.08	0.299
Interaction: TA × SA	**6.49**	**0.002**	1.55	0.213	1.56	0.210	0.33	0.723
Interaction: AC × TA	0.02	0.981	1.58	0.208	0.27	0.764	0.19	0.829
Interaction: AC × SA	0.71	0.401	0.96	0.328	0.50	0.480	0.01	0.936


*ANOVA on the ipsi-lateral BOLD signal from icnV1 and icnV2 at time points PRE-stimulus (PRE) and PEAK-response (PEAK) including the condition ‘access to consciousness’ and the two attention conditions. Degrees of freedom (df), *F*-values, and *p*-values are reported. Given *p*-values are not corrected. *p*-values significant at a Bonferroni-correction for four comparisons are highlighted in bold.*

We next tested whether the ipsi-lateral BOLD magnitude was influenced by our attentional manipulations. At the time of PEAK-response, BOLD activity in icnV1 and icnV2 was affected by temporal attention, with higher percent signal change at shorter cue-target SOAs (icnV1: *p* < 0.001, icnV2: *p* < 0.001, Bonferroni-corrected). Importantly, there were no significant interactions or trends for interactions in the BOLD magnitude between consciousness categories and any of the attentional conditions (**Table 1**). Our main findings did thus not depend on particular attentional configurations.

Mixed-effects analyses, which were performed in parallel, were in agreement though less significant. The following results were obtained with regard to BOLD magnitude differences between consciousness categories: (i) V1 PRE-stimulus *t* = 2.31, *p* = 0.021, (ii) V2 PRE-stimulus *t* = 2.72, *p* = 0.007, (iii) V1 PEAK-response *t* = -1.78, *p* = 0.076, and (iv) V2 PEAK-response *t* = -0.40, *p* = 0.693. Explicitly testing for a BOLD magnitude increase in icnV1 for conscious target perception (see Schölvinck et al., 2012) resulted in a significant effect (one-tailed; V1 PEAK-response *t* = -1.78, *p* = 0.038). All df = 907. *p*-values are not Bonferroni-corrected.

### Association between Contra-Lateral BOLD Magnitude and Visual Consciousness

We subsequently examined the relationship between the BOLD magnitude in stimulated ROIs of the contra-lateral hemisphere and conscious visual perception. Corresponding *F*- and *p*-values are assembled in **Table 2**. As can be seen in **Figures 4C,D**, the BOLD magnitude at the time of PEAK-response was higher for consciously perceived targets, both in icnV1 and in icnV2. This relation was statistically significant (icnV1: *p* = 0.011; icnV2: *p* = 0.003, Bonferroni-corrected). During stimulation, conscious visual perception was thus clearly associated with the BOLD magnitude in the contra-lateral ROIs.

**Table 2:** Contra-lateral BOLD activity in V1 and V2.

	Contra-V1 (df = 390)				Contra-V2 (df = 724)			
		
	PRE		PEAK		PRE		PEAK	
				
	*F*	*p*	*F*	*p*	*F*	*p*	*F*	*p*
Access to consciousness (AC)	1.05	0.307	**9.12**	**0.003**	0.27	0.606	**11.23**	**0.001**
Temporal attention (TA)	1.49	0.227	2.70	0.069	2.71	0.068	**5.54**	**0.004**
Spatial attention (SA)	2.31	0.129	0.13	0.723	0.08	0.772	0.60	0.440
Interaction: TA × SA	0.02	0.985	0.10	0.907	1.30	0.273	2.58	0.077
Interaction: AC × TA	0.23	0.792	1.27	0.282	0.30	0.740	2.99	0.051
Interaction: AC × SA	0.11	0.740	1.18	0.279	0.23	0.633	0.37	0.542


*ANOVA on the contra-lateral BOLD signal from icnV1 and icnV2 at time points PRE-stimulus (PRE) and PEAK-response (PEAK) including the condition ‘access to consciousness’ and the two attention conditions. Degrees of freedom (df), F-values, and *p*-values are reported. Given *p*-values are not corrected. *p*-values significant at a Bonferroni-correction for four comparisons are highlighted in bold.*

Regarding our attentional manipulations, there was a significant effect of temporal attention on the BOLD signal change in the contra-lateral icnV2 at the time of PEAK-response (*p* = 0.016, Bonferroni-corrected), with higher BOLD magnitudes at shorter cue-target SOAs. At the site of stimulus processing in the first higher-tier area V2, uncorrected *p*-values (**Table 2**) indicate a trend in the interaction of access to consciousness and temporal attention after target presentation (Bonferroni-corrected: *p* = 0.204). This might be explained by stimulus processing within contra-lateral V1, which experiences influences from ongoing fluctuations as well as attention manipulation at the same time.

### PRE-Stimulus Ipsi-Lateral Activity is Associated with PEAK-Response Contra-Lateral Activity

**FIGURE 5:**
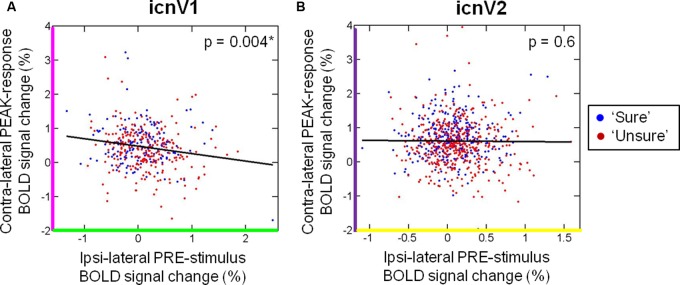
**Relation between contra-lateral and ipsi-lateral blood oxygenation level dependent (BOLD) percent signal change.** The correlation of the contra-lateral PEAK-response signal and the ipsi-lateral PRE-stimulus signal is shown for **(A)** icnV1 and **(B)** icnV2. Blue dots refer to trials of the category ‘sure’, whereas red dots indicate trials of the category ‘unsure’. *p*-values were calculated from *t*-tests across participants on Fisher-Z-transformed single-subject correlation coefficients. Significant *p*-values are marked by ^∗^.

We further investigated whether ongoing activity, approximated by the ipsi-lateral BOLD signal before stimulation, influenced the percentage of contra-lateral BOLD signal change during stimulation. To do so, we calculated the correlation of ipsi-lateral PRE-stimulus activity with contra-lateral PEAK-response activity. As depicted in **Figure 5**, PRE-stimulus ipsi-lateral activity correlated significantly with PEAK-response contra-lateral activity in icnV1 (R: median = -0.13, Fisher-Z transformed R: mean ± standard deviation = -0.15 ± 0.12, *p* = 0.004, *N* = 10), but not in icnV2 (R: median = 0.05, Fisher-Z transformed R: mean ± standard deviation = 0.03 ± 0.21, *p* = 0.630, *N* = 15). This correlation was negative, as expected from the reduced PRE-stimulus ipsi-lateral activity and increased PEAK-response contra-lateral activity in icnV1 associated with conscious visual perception (see **Figure 4**).

### The Phase of Ipsi-Lateral Slow BOLD Fluctuations before Target Presentation is Associated with the Access to Visual Consciousness

**FIGURE 6:**
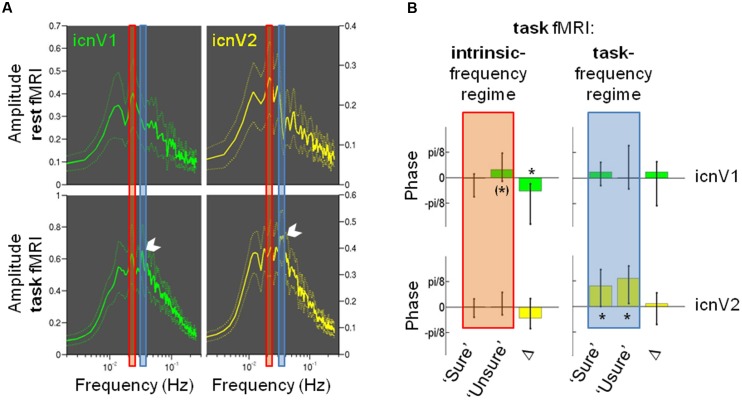
**The relevance of signal phases for visual consciousness.** **(A)** Amplitude spectra as mean and standard error of the mean for ipsi-lateral icnV1 (green) and ipsi-lateral icnV2 (yellow), calculated from the whole-brain resting-state run (upper panels) and the task runs (lower panels). The peak corresponding to the task stimulation frequency is highlighted in the lower panels by a white arrow. Also highlighted are the regimes of intrinsic frequency (red) and task frequency (blue), which were used for further calculations of frequency-specific phases. **(B)** Phase values (median and 50% confidence intervals) prior to target presentation in the task runs for ipsi-lateral icnV1 (green) and icnV2 (yellow), separately for each frequency regime (intrinsic: red, task: blue) and each consciousness category (‘Sure,’ ‘Unsure’). Δ illustrates the median and 50% confidence interval of the individual phase differences between consciousness categories. Phases φ range from -π to π and translate into signal by φ = 0 meaning signal peak and φ = π or -π meaning signal trough. Significant deviations from an average phase of zero and non-zero Δ are marked by ^∗^, trends by ^(∗)^.

**Figure 6** shows results of a frequency-based analysis of the BOLD signals from ipsi-lateral icnV1 and icnV2. Spectra from task data are depicted along with spectra from a whole-brain resting-state fMRI measurement. The task data revealed a clear fingerprint of the task in icnV1 and icnV2 activity as illustrated by the white arrow in **Figure 6A**. The stimulation frequency of 0.0334 Hz was associated with the highest spectral amplitude in both ICNs. In contrast, spectra of fMRI data acquired under resting-state conditions prior to the task runs revealed that the highest amplitude for ongoing BOLD activity was located at lower frequencies (0.0234 Hz for icnV1 and 0.0215 Hz for icnV2). In icnV1, this peak was preserved within the task data spectrum. In icnV2, however, the task spectrum did not contain a similarly pronounced peak within the ‘intrinsic-frequency regime’ as at rest. There were thus clear spectral differences between ipsi-lateral activity during task and rest.

Phases before target presentation were therefore assessed separately for the ‘intrinsic-’ and the ‘task-frequency regime’ as well as for ipsi-lateral icnV1 and icnV2 and the two consciousness categories (see **Figure 6B**). Regarding the ‘task-frequency regime,’ phases in both consciousness categories did not deviate from a mean value of 0 in icnV1 (‘sure’: *p* = 0.421, ‘unsure’: *p* = 0.535, *N* = 16, Wilcoxon signed rank test), while they took significantly positive values in icnV2, representing periods of predominant signal decay (‘sure’: *p* = 0.008, ‘unsure’: *p* = 0.011, *N* = 16, Wilcoxon signed rank test). In neither of the ICNs within the ‘task-frequency regime’ did the average phase value differ significantly with respect to the consciousness category (icnV1: *p* = 0.449, icnV2: *p* = 0.368, *N* = 16, Wilcoxon signed rank test), suggesting that the previously described effects of BOLD magnitude differences did not originate from this frequency regime.

Within the ‘intrinsic-frequency regime’, there was a trend for a predominant signal decrease, expressed by positive phase values, in the case of the ‘unsure’ category in icnV1 (*p* = 0.056, *N* = 16, Wilcoxon signed rank test). Phases of the ‘sure’ category in this ICN on the other hand did not deviate significantly from a mean value of 0 (*p* = 0.489, *N* = 16, Wilcoxon signed rank test). Similarly, there was no significant deviation from 0 in any of the consciousness categories in icnV2 (‘sure’: *p* = 0.847, ‘unsure’: *p* = 0.535, *N* = 16, Wilcoxon signed rank test). We finally examined whether phases of the ‘intrinsic-frequency regime’ differed significantly between the ‘sure’ and the ‘unsure’ category in any of the ICNs. In icnV1, the difference in the phase values for the two consciousness categories was significant (icnV1: *p* = 0.030, icnV2: *p* = 0.197, *N* = 16, Wilcoxon signed rank test). This analysis therefore suggests that the observed BOLD magnitude difference between consciousness categories leaves out the ‘task-frequency regime’ and its harmonics and on the contrary resides in the ‘intrinsic-frequency regime.’

Importantly, there were no significant differences between intrinsic phase values of the different attention conditions, neither in icnV1 (temporal attention: *p* = 0.877 (2–4 s), *p* = 0.278 (2–6 s), *p* = 0.234 (4–6 s), spatial attention: *p* = 0.469, *N* = 16, Wilcoxon signed rank tests) nor in icnV2 (temporal attention: *p* = 0.679 (2–4 s), *p* = 0.877 (2–6 s), *p* = 0.959 (4–6 s), spatial attention: *p* = 0.196, *N* = 16, Wilcoxon signed rank tests). The lack of statistically detectable differences in this exploratory analysis further substantiates the absence of task effects in our ongoing BOLD signal.

## Discussion

The present study provides evidence that bilateral ongoing BOLD activity, approximated by fluctuations in ipsi-lateral parts of intrinsic occipital networks, is strongly associated with an incoming stimulus’ access to visual consciousness. In particular, consciously perceived stimuli were characterized by a significantly higher ipsi-lateral BOLD magnitude after stimulus presentation in V1. Phase analyses revealed that this effect was most likely caused by stronger declines of the ongoing BOLD signal previous to stimulation in the case of non-conscious stimuli. Finally, our results highlight the role of intrinsic networks covering V1 as opposed to those covering V2 with respect to conscious visual perception.

### Relation between Ipsi-Lateral BOLD Magnitude in V1 and the Access to Consciousness

Our results are in line with previous studies exploring signal fluctuations in non-stimulated parts of an ICN and their relation to performance (Fox et al., 2007; Schölvinck et al., 2012). All of these studies indicate that ongoing BOLD activity accounts for trial-to-trial differences in the BOLD magnitude at the site of direct task stimulation. Such differences in turn can be associated with task measures such as conscious visual perception (see **Figure 4**). As these findings reflect trial-to-trial variability in response to identical stimuli, they cannot be explained by task-evoked differences in BOLD activity. The variability over and above task-locked effects therefore is likely to reflect intrinsic, ongoing processes. In our case, none of the systematic variations on single trials did interact with this effect. Our results finally go beyond previously published accounts by providing evidence that processes underlying the pre-stimulus ipsi-lateral BOLD signal bear importance for the subsequent access to visual consciousness.

Leaving the left hemisphere without direct stimulation and defining ROIs both in an intrinsic network-wide way and independently from the experiment proper (**Figure 2**) offered the possibility to characterize proxies for ongoing activity of the entire ICNs V1 and V2 and thus to avoid influences of focal effects such as attentional top-down effects on specific retinotopic positions (Tootell et al., 1998a; Brefczynski and DeYoe, 1999; Saygin and Sereno, 2008). Yet, one might argue that our proxies for ongoing BOLD activity were not completely free of task effects. In line with previous findings, there was indeed a clear imprint of the task in the signal we used (Tootell et al., 1998b; Smith et al., 2004). This imprint is depicted in **Figures 4A,B** as a BOLD signal drop after target presentation and results in the task-related peaks in **Figure 6A**. **Figure 6B** confirms this finding by showing a dominance of decreasing phases in the ‘task-frequency regime’ of icnV2. Such an effect has previously been interpreted as a consequence of attentional shifts to the stimulated hemisphere or alternatively as blood stealing (Tootell et al., 1998b; Smith et al., 2004). As the target in our task was preceded by a cue, the described effect could possibly reflect a reaction to this cue event (Ress et al., 2000) within the ‘task-frequency regime.’ Importantly, no comparable task imprints were observed in the ‘rest-frequency regime,’ indicating that our main results can indeed be attributed to ongoing BOLD activity.

Finally, it cannot be excluded that top–down feedback of the elicited consciousness-related processes on the time scale of milliseconds caused the magnitude difference between the two consciousness categories. The putative increased relevance of top–down feedback in V2 as compared to V1, however, renders this explanation less likely (e.g., Melloni et al., 2012).

### Relation between Ipsi-Lateral BOLD Phase in V1 and the Access to Consciousness

To our knowledge, this is the first study describing a relation of ongoing BOLD signal phases and a performance measure. Our study suggests that predominant signal decay prior to stimulation is indicative of failed access to consciousness (**Figure 6B**, left panel). The influence of the task paradigm on this phase-performance relation can be ruled out because band-pass filtering was employed to the regime of ongoing BOLD fluctuations at rest. Phase effects of this kind are well known from electrophysiology studies (Devrim et al., 1999). Furthermore, these findings are in agreement with our results on BOLD magnitudes. High BOLD magnitudes seconds before stimulation tended to decline prior to the target and were consequently associated with a reduced likelihood of conscious awareness. During the chosen period of observation, the signal thus evolved qualitatively differently depending on the subsequent access to consciousness. Notably, this oscillatory signal was of frequencies below 0.08 Hz, which exactly characterize the dynamics of ongoing BOLD fluctuations (Fox et al., 2005; Fox and Raichle, 2007).

BOLD fluctuations within ICNs have been directly linked to electrophysiological SCPs (Hiltunen et al., 2014). Both signals are thought to be relevant for the coordination of activity states between more distant brain regions, possibly by modulating local high-frequency processes via phase-amplitude coupling (Khader et al., 2008; He and Raichle, 2009; Thompson et al., 2014). Interestingly, He and Raichle proposed that both BOLD and SCP fluctuations in the visual system contribute critically to processes of visual consciousness (He and Raichle, 2009). Several studies indeed report correlations between the SCP phase and behavioral performance (Birbaumer et al., 1990; Devrim et al., 1999; Monto et al., 2008). In particular, Devrim et al. (1999) found a significantly increased visual detection rate during states with negative as opposed to positive cortical potential shifts in the range of SCPs at occipital electrodes. Our data provide further evidence for this model by linking the phase of slow BOLD fluctuations in V1 directly to conscious visual perception.

Regarding the translation of electrophysiological effects to fMRI results, a sluggishness of the BOLD response in the range of seconds has to be taken into account. Further studies have to clarify whether a falling BOLD signal, as detected in our ‘unsure’ category, could be reflecting a rising SCP amplitude.

### Relative Roles of V1 and V2 for the Access to Consciousness

We detected a higher relevance of ipsi-lateral BOLD fluctuations within V1 as opposed to V2 for the access to visual consciousness. In line with these findings, previous studies point at V1 as an important prerequisite for conscious visual experience (Crick and Koch, 1995; Tong, 2003; Persaud and Lau, 2008; Weiskrantz, 2009; Ffytche and Zeki, 2011; Aru et al., 2012; Leopold, 2012). Tse et al. (2005), for example, found an elevated BOLD signal throughout V1 (and V2 to a lesser extent) to be related to visual awareness in a masking paradigm, though they did not compare stimulated and non-stimulated sites. With regard to the significant link between ongoing ipsi-lateral and task-evoked contra-lateral activity in V1 (see **Figure 5A**), our results correspond well with other studies suggesting a contribution of ongoing fluctuations to visual task-evoked BOLD activity in early visual areas (Arieli et al., 1996; Bianciardi et al., 2009; Cardoso et al., 2012; Schölvinck et al., 2012).

### Methodological Considerations

Finally, four methodological considerations regarding our experimental design and data analyses should be mentioned.

(i) Operationalization of consciousness. The access to consciousness in our study was operationalized by means of subjective ratings (Dehaene and Changeux, 2011). Within a two-point rating scale, participants were asked whether they were sure or guessed the answer (Lau and Passingham, 2006). These ratings thus involved confidence as a measure for consciousness. As states in which participants had a “weak glimpse” (Andersen et al., 2016) of the target were most likely attributed to the ‘unsure’ category, the rate of correct responses in this category was increased above chance level. A finer grading in further studies could improve event classification and attribution of occipital activity to the different consciousness categories.
(ii) Definition of ongoing BOLD activity. As discussed above, the present study used an aggregate signal from non-stimulated ipsi-lateral parts of occipital ICNs as the proxy for ongoing BOLD activity. This assures that a substantial part of those regions is considered which routinely interact within the bilateral ICNs but receive no direct stimulation. A number of studies certify an imprint of a visual task in the ipsi-lateral part of the visual cortex (Tootell et al., 1998b; Smith et al., 2004; Sylvester et al., 2007; Chang et al., 2012; He, 2013). Within our study, task effects have been controlled for by (i) explicitly focusing on trial-to-trial variability, (ii) ruling out interaction effects of the task paradigm’s attentional conditions, and iii) concentrating on the ‘intrinsic-frequency regime’ while also analyzing the ‘task-frequency regime,’ which yielded qualitatively different results.
(iii) Statistical analysis of BOLD magnitudes. As the BOLD percent signal change in a primary sensory cortex such as the occipital cortex is assumed to be comparable across participants, we used a fixed-effects model for group analyses. However, no population inferences can be made from such a model and future studies are needed to examine whether these inferences are possible. An additional mixed-effects model analysis provided supporting results.
(iv) The applied criterion for exclusion of trials due to eye movements was generous, but allowed for exclusion of eye blinks and assured participants did not purposefully or accidentally observe the target position. Data analysis was performed on an averaged signal within the whole areas of ipsi-lateral icnV1 and ipsi-lateral icnV2 (more than 6 mm away from the inter-hemispheric gap). This assured a certain degree of robustness towards regional effects in single events when fixation would not be absolute. The additional analysis in small spherical contra-lateral ROIs showed that significant activations were elicited on distinct sites in retinotopic contra-lateral icnV1 and icnV2. This serves as confirmation that the stimulation happened at specific sites of the retina and that eye movements were minor within the included events.

## Conclusion

In summary, our results provide evidence for the significant and specific relation of slowly fluctuating ongoing V1 activity and the emergence of visual consciousness.

## Author Contributions

AW, VR, and CS were involved in the conception and design of the study. AW, JD, LK, VR, and CS developed the methods. JD, LK, VR, and CS acquired the data. AW, SG, JS, JD, LK, and SL performed the analyses. AW, SG, VR, and CS wrote the manuscript.

## Conflict of Interest Statement

The authors declare that the research was conducted in the absence of any commercial or financial relationships that could be construed as a potential conflict of interest.

## Footnotes

1 https://www.neurobs.com

2 http://www.mrivideo.com

3 http://www.arringtonresearch.com

4 http://www.fil.ion.ucl.ac.uk/spm/

5 http://icatb.sourceforge.net

6 http://www.mathworks.de/
